# Endoscopic submucosal dissection for residual gastric lesion made easier thanks to use of adaptative traction device

**DOI:** 10.1055/a-2286-5543

**Published:** 2024-04-03

**Authors:** Elena De Cristofaro, Pierre Lafeuille, Jérôme Rivory, Jean Grimaldi, Louis Jean Masgnaux, Alexandru Lupu, Mathieu Pioche

**Affiliations:** 1 9318 Gastroenterology Unit, University of Rome Tor Vergata, Rome, Italy; 2 Gastroenterology and Endoscopy Unit, Edouard Herriot Hospital, Hospices Civils de Lyon, Lyon, France

European guidelines recommend endoscopic submucosal dissection (ESD) for gastric dysplastic lesions to ensure en bloc resection and for a lower risk of recurrence than endoscopic mucosal resection (EMR) 1 . However, this procedure is considered technically challenging especially for residual lesions in which the fibrosis after previous treatments can be found in the submucosal space and EMR is still performed with a local risk of recurrence. Herein, we report the case of a 66-year-old woman assessed for ESD of a residual dysplastic gastric lesion at the site of previous piecemeal EMR resection (posterior wall of antrum) ( Video 1 ).

Adaptive traction device makes endoscopic submucosal dissection for residual gastric lesion easier.Video 1

Several techniques, such as traction strategies, have already been described to facilitate these technically challenging procedures. In previous cases we reported the benefits of using a new adaptative multipolar traction system (A-TRACT) in different challenging resections. This device facilitates the dissection phase through exposing the submucosa and accelerating the procedure 2 3 4 5 .

After circumferential incision with large margins and submucosal trimming, an adaptative traction device (A-TRACT 2) was used to improve submucosal exposure ( Fig. 1 ). The two loops were fixed by clips at the edges of the lesion. The rubber band was fixed to the opposite wall to achieve 90° of traction and the dissection was started with traction. The device was tightened when exposure was poor, and the procedure was completed after 35 minutes with good submucosal exposure and no adverse events. Resection was R0 and the histopathology revealed an adenoma with low-grade dysplasia.

**Fig. 1:**
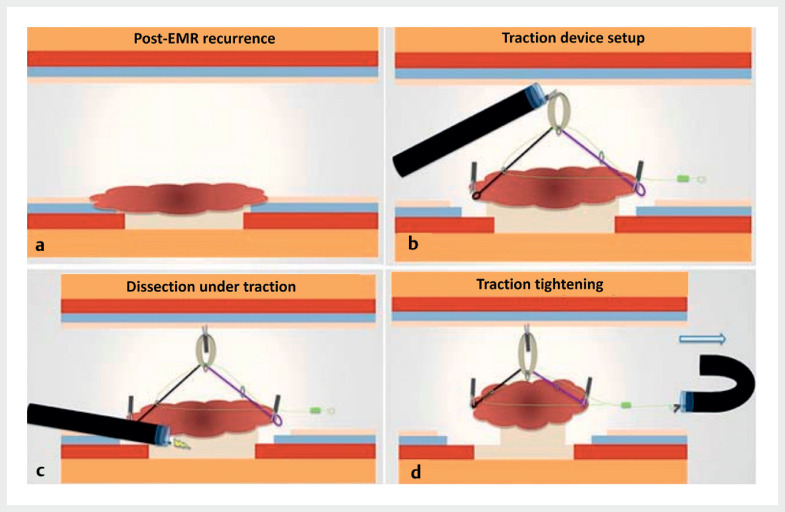
Schematic view after piecemeal endoscopic mucosal resection (EMR). **a** EMR recurrence. **b** Traction device setup. **c** Dissection under traction. **d** Device tightening to improve traction.

We can assume that this adaptative traction device can make ESD procedures feasible and faster, especially in selected cases like residual lesions, allowing curative endoscopic treatment.

Endoscopy_UCTN_Code_TTT_1AO_2AG_3AD

## Notes

Endoscopy E-Videos https://eref.thieme.de/e-videos
*E-Videos* is an open access online section of the journal *Endoscopy* , reporting on interesting cases and new techniques in gastroenterological endoscopy. All papers include a high-quality video and are published with a Creative Commons CC-BY license. Endoscopy E-Videos qualify for HINARI discounts and waivers and eligibility is automatically checked during the submission process. We grant 100% waivers to articles whose corresponding authors are based in Group A countries and 50% waivers to those who are based in Group B countries as classified by Research4Life (see: https://www.research4life.org/access/eligibility/ ). This section has its own submission website at https://mc.manuscriptcentral.com/e-videos .
